# Classification of *Camellia oleifera* Diseases in Complex Environments by Attention and Multi-Dimensional Feature Fusion Neural Network

**DOI:** 10.3390/plants12142701

**Published:** 2023-07-20

**Authors:** Yixin Chen, Xiyun Wang, Zhibo Chen, Kang Wang, Ye Sun, Jiarong Jiang, Xuhao Liu

**Affiliations:** 1 School of Information Science and Technology, Beijing Forestry University, No. 35 Qinghuadong Road, Beijing 100083, China; frizen@bjfu.edu.cn (Y.C.); wxy1120@bjfu.edu.cn (X.W.); kangkangkang@bjfu.edu.cn (K.W.); sunye0206@bjfu.edu.cn (Y.S.); jothjiang@bjfu.edu.cn (J.J.); 2 Engineering Research Center for Forestry-Oriented Intelligent Information Processing of National Forestry and Grassland Administration, Beijing 100083, China; 3 School of Biological Sciences and Biotechnology, Beijing Forestry University, No. 35 Qinghuadong Road, Beijing 100083, China; liuxuhao@bjfu.edu.cn

**Keywords:** *Camellia oleifera* diseases classification, convolutional neural networks, attention mechanism, feature fusion

## Abstract

The use of neural networks for plant disease identification is a hot topic of current research. However, unlike the classification of ordinary objects, the features of plant diseases frequently vary, resulting in substantial intra-class variation; in addition, the complex environmental noise makes it more challenging for the model to categorize the diseases. In this paper, an attention and multidimensional feature fusion neural network (AMDFNet) is proposed for *Camellia oleifera* disease classification network based on multidimensional feature fusion and attentional mechanism, which improves the classification ability of the model by fusing features to each layer of the Inception structure and enhancing the fused features with attentional enhancement. The model was compared with the classical convolutional neural networks GoogLeNet, Inception V3, ResNet50, and DenseNet121 and the latest disease image classification network DICNN in a self-built camellia disease dataset. The experimental results show that the recognition accuracy of the new model reaches 86.78% under the same experimental conditions, which is 2.3% higher than that of GoogLeNet with a simple Inception structure, and the number of parameters is reduced to one-fourth compared to large models such as ResNet50. The method proposed in this paper can be run on mobile with higher identification accuracy and a smaller model parameter number.

## 1. Introduction

*Camellia oleifera* Abel., also known as oil tea, is a small evergreen tree that is one of the world’s four major woody oil trees. Research indicates that camellia oil contains a comparable amount of fatty acids to olive oil. Long-term consumption of camellia oil has been shown to aid in the treatment of cardiovascular diseases, lower cholesterol levels, and protect the liver [1,2]. Furthermore, camellia oil also exhibits properties that enhance antioxidation and anti-inflammation, while regulating the gut microbiota and its metabolites. It can alleviate ethanol-induced gastric mucosal damage and significantly reduce the risk of gastric ulcers [3]. The husks of *C. oleifera* seeds also hold significant practical value. The water-soluble polysaccharides found in the husks exhibit potent antioxidant properties, making them potential safe anticancer agents for industrial applications [4]. It can also be used as an alternative raw material for the manufacture of chipboard [5]. Nevertheless, disease reports concerning *C. oleifera* highlight its vulnerability to 51 pathogens, encompassing fungi, bacteria, parasitic plants, nematodes, lichens, and mosses, among others. The proliferation of these pathogens leads to a wide array of diseases that spread extensively throughout plantations, posing a significant threat to the overall quality of *C. oleifera* products [6].

Artificial intelligence has advanced at a breakneck pace in recent years, making waves in fields like picture classification and recognition, as well as being widely used in the field of crop disease diagnosis, which has become a major trend in plant disease identification [7]. Specific techniques include machine learning, deep learning, and attention mechanism. Traditional machine learning algorithms perform disease classification by utilizing mathematical indicators such as the area and number of disease spots. This approach necessitates intricate preprocessing of the original image, resulting in substantial cost implications. Furthermore, plant diseases are diverse and can occur in clusters, making feature extraction more complex and potentially leading to erroneous recognition findings. Deep learning, which can automatically extract picture features through backpropagation and reduces the difficulty of extraction, is therefore often utilized in agricultural weed identification and disease detection tasks [8,9,10]. Sladojevic et al. [11] first proposed using convolutional neural networks (CNNs) for disease detection and classification of plant leaves. They used five convolutional and three fully connected layers for a total of eight learning layers to form pre-trained convolutional neural networks and fine-tuned them on a self-built dataset. The average accuracy of the experimental results was 96.3%. By increasing the depth of CNN convolutional layers, Selvi et al. [12] created a deep convolutional neural network architecture for classifying weeds and crops. On a dataset of sesame crops with a variety of weeds, the classification accuracy was 95%. Liu et al. [13] used GoogLeNet and DenseNet to create a Dense Inception Convolutional Neural Network (DICNN), which allows feature propagation and reuse by densely linking Inception modules to solve the gradient disappearance issue. This improved the extraction performance of multi-scale disease patches. The experimental results reveal that DICNN surpasses other popular migration learning models for seven grapevine disease leaves, with an accuracy of 97.22%. When compared to standard ResNet and GoogLeNet, the convergence speed and accuracy are increased. Bruno et al. [14] configured several weak models in parallel and adaptively integrated them by CNN. By using a trainable layer to fuse feature vectors instead of aggregating outputs, the performance of the weak model can match the performance of the complex model and even SOTA on PlantVillage. 

Nevertheless, deep learning has trouble extracting the needed features when there are slight differences between object kinds and huge differences within types, making it easy to draw inaccurate conclusions [15]. In addition, the presence of background noise in the real environment makes it more difficult for image features to be extracted accurately [16]. Therefore, the researchers solved this problem by simulating the formation of attention in the human brain and assigning weights to different features. 

Previously, deep learning models focused on the entire input image; however, with the introduction of attention mechanisms, deep learning models may now focus on specific regions in the input image, enabling them to focus on local features [17]. Attention mechanism has grown in prominence in the field of deep learning in recent years, and are now utilized in a variety of tasks such as natural language processing, picture segmentation, and fine-grained categorization. The attention mechanism can also be used in several steps in image classification, and the most typical application is to reinforce the image features that the CNN has already extracted, improving the extraction of fine features with differentiation. Zhao et al. [18] solved the network degradation problem by merging the residual structure into the Inception structure. They also include the convolutional block attention module (CBAM) in the feature extraction layer, replacing the Multi-Layer Perceptrons (MLP) in CBAM with two one-dimensional convolutions to capture cross-channel information and prevent picture feature corruption. The upgraded convolutional neural network performs well in classifying plant leaf diseases. In the corn, potato, and tomato datasets that were taken from the PlantVillage dataset, the model achieved an overall accuracy of 99.55%. Zhao et al. [19] proposed DTL-SE-ResNet50 based on ResNet50 by combining Squeeze and Excitation Module (SENet) for feature map optimization and dual migration learning. On a dataset of vegetable leaf diseases, an accuracy rate of 97.24% was attained, outperforming the conventional classification network. When compared to DTL-SA-ResNet50, which uses spatial attention, and DTL-CBAM-ResNet50, which uses the CBAM module, the three modules are similar for model optimization, but the SENet inference time will be considerably shorter. Pandey and Jain [20] proposed the attentional residual learning (ARL) mechanism by combining the attentional learning mechanism with the residual learning block. In a dataset created from 15 classes of plant health images, the network achieved a 98.20% accurate classification rate using discriminative feature extraction from RGB plant leaf images via ARL. In addition, there are also attempts to add the attention mechanism to the classification module of convolutional neural networks to improve the correct classification rate by assigning weights to different feature maps. Turkoglu et al. [21] used a Long Short-Term Memory (LSTM) network to pay attention to the inputs of the fully connected layers of the AlexNet, GoogLeNet, and DenseNet201 models, adding attention to the classification layer of the model. The final output is then decided by majority voting. It has increased the accuracy rate by 10% when compared to the traditional Support Vector Machine (SVM) classifier. 

Since the image features were extracted by superimposing layers of convolutional kernels in convolutional neural networks, the receptive field of the convolutional kernels of different layers is different, and the fineness of the extracted features is also different. Generally speaking, the bottom feature map has less semantic information, but the target location is accurate; the top feature map has richer semantic information, but the location is coarser [22]. Due to this, it is difficult to classify some diseases with small vision feature using the conventional classification approach that uses only top features. To improve the extraction of picture features and fully utilize the semantic information of distinct layers, researchers suggest feature fusion strategies to aggregate features comprising diverse semantic information to produce more reliable and accurate recognition results. Lin et al. [22] upsampled and scaled the higher-level features of the neural network, and features corresponding to the bottom-level feature elements were summed to propose Feature Pyramid Networks (FPN). FPN fuses the higher-level and bottom-level features while allowing multi-scale prediction and adapting to targets of different scales, which is more effective than using single-layer features directly. Dai et al. [23] proposed the Attention Feature Fusion (AFF) structure by using local channel attention and global channel attention, calculating the weights between input features, and fusing the input features based on the weights. AFF can better fuse the semantics and solve the scale discontinuity problem. Experiments showed that adding the AFF structure to the Inception, Resnet, and FPN structures can effectively improve the correct rate. By integrating deep features with histogram of oriented gradients (HOG) features in channels, Fan et al. [24] improved the local spatial characteristics in images of plant leaves. On the apple leaf dataset, an accurate rate of 93.19% was attained, which is 1.91% higher than the model without fusion. 

This study suggests a *C. oleifera* disease recognition model utilizing attention mechanism and multidimensional feature fusion in order to address the issues of low recognition accuracy, the significant influence of complex background, and large model computation, and to achieve fast and accurate recognition of diseases. Based on the Inception structure, the model was optimized by utilizing attention mechanism and feature fusion techniques. The new model was trained using data from a self-built database of frequent *C. oleifera* leaf diseases. The model’s running time, recognition accuracy, recall, precision, number of parameters, and floating points of operations (FLOPs) were applied as evaluation criteria for *C. oleifera* diseases recognition technology. 

Based on this study and analysis shown above, this paper presents a method for classifying *C. oleifera* diseases using multidimensional feature fusion and an attention mechanism, and the model is evaluated and compared. Section 2 describes the process of gathering and creating the *C. oleifera* disease dataset, as well as the concepts and methods used in the *C. oleifera* disease classification model. Section 3 details the experimental environment and experiment design, as well as a comparative analysis of the outcomes. Section 4 summarizes the experimental data analysis and presents a forecast for the future. In Section 5, conclusions are drawn and prospects are discussed.

## 2. Results

### 2.1. Experiment Setup

The models in this study were tested on CentOS 7.4 using the PyTorch framework, accelerated in parallel by four Tesla T4 GPUs with 16 GB of graphics memory. The performance of classical models, such as VGG19, GoogLeNet, InceptionV3, ResNet50 and DenseNet121, the latest plant disease classification models, such as DICNN, and the proposed AMDFNet were compared on a self-built *C. oleifera* disease dataset. All models were pre-trained on the ISLVRC2012 dataset in order to improve the training accuracy and reduce the number of training sessions.

**Figure 1:**
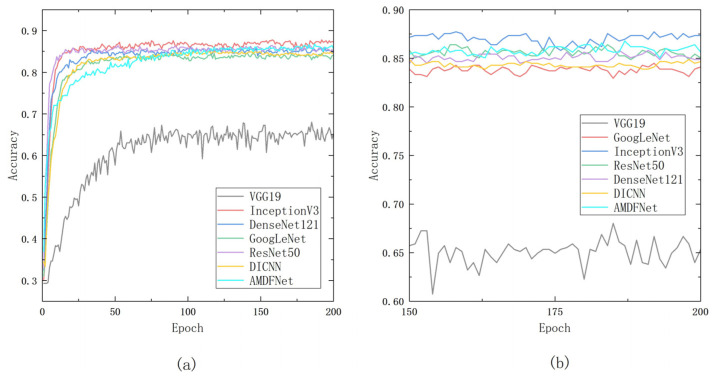
Accuracy of the validation set curves in the training process: (**a**) accuracy curves throughout the process; (**b**) accuracy curves in the last fifty epochs.

On the ISLVRC2012 dataset, all models were trained with a batch size of 256 and a learning rate of 0.01 over 200 rounds. In order to save time, the early-stop strategy was used during the pre-training, and the training was stopped when the validation set loss did not decrease for 10 consecutive rounds. For migration learning, the batch size is set to 256, the learning rate is set to 0.001, and when the quantity of training rounds exceeds 30, 70, and 160 rounds, the learning rate is decreased to one-tenth of the previous one. The accuracy curve of the validation set is shown in Figure 1.

### 2.2. Model Evaluation Indicators

Convolutional neural networks have numerous parameters and a wide range of applications, so there are many indicators that can be used to evaluate the model’s performance. Considering the target audience of the model and the application environment, in this study, the time of a single image, parameter size, accuracy, recall, and precision were selected to evaluate the *C. oleifera* disease identification model.

#### 2.2.1. Time of a Single Image (*T_s_*)

One important parameter for model evaluation is the processing time of a single image. Short model training times not only speed up model training and make parameter learning easier, but they also increase the effectiveness of mobile application identification. To make model comparison easier, the time needed to evaluate an image can be estimated as stated in Equation (1).

(1) $$T_{s}=\frac{Total\;test\;time}{Total\;number\;of\;test\;images}$$

#### 2.2.2. Parameter Size (*Params*)

The number of parameters is the smallest amount of computer memory that must be requested while the program runs, and it serves as a thorough evaluation indicator of the model’s overall performance, which is calculated as shown in Equation (2).

 *param* = *param_conv_* + *param_f c_*
      
*param_conv_* = (*k_w_* + *k_h_* + *c_in_*) × *c_out_* + *c_out_*
 *param_f c_* = *c_in_* × *c_out_* + *c_out_* (2)

where *param_conv_* and *param_f c_* represent the number of parameters in the convolutional and fully connected layers, respectively, *k* represents the width and height of the kernel, and *c* represents the number of channels.

The quantity of parameters impacts both the minimum hardware needed for model operation and the speed of operation. As a result, a key criterion for assessing the model in this study is the number of parameters.

#### 2.2.3. Floating Points of Operations (*FLOPs*)

The FLOPs measure the computational complexity of the model by calculating the number of multiplication and addition operations in the model. The higher the FLOPs, the more computationally intensive the model is, which means the higher the hardware requirements such as graphics cards. The number of floating point operations in the fully connected layer is only related to the number of parameters, whereas the number of floating point operations in the convolutional layer is also related to the size of the input feature map, as shown in Equation (3).

*FLOPs* = *FLOPs_conv_* + *FLOPs_f c_*
           *FLOPs_conv_* = [2 × (*k_w_* × *k_h_* × *c_in_*) × *c_out_* + *c_out_*] × *H* × *W*

 *FLOPs_f c_* = 2 × (*c_in_* × *c_out_*) + *c_out_* (3)

#### 2.2.4. Accuracy (*Acc*)

The accuracy is the percentage of categories properly predicted by the model out of the entire data volume, as shown in Equation (4).

(4) $$A_{CC}=\frac{T_{P}+T_{N}}{T_{P}+T_{N}+F_{P}+F_{N}}\times 100\%$$

where *T_P_* denotes the number of true positives, *T_N_* denotes the number of true negatives, *F_P_* denotes the number of false positives, and *F_N_* denotes the number of false negatives.

#### 2.2.5. Recall (*R*)

Recall represents the probability that a diseased sample is detected in each category and is used to reflect the disease detection capability of the model. Recall is calculated as shown in Equation (5).

(5) $$R=\frac{T_{P}}{T_{P}+F_{N}}\times 100\%$$

where *T_P_* denotes the number of true positives and *F_N_* denotes the number of false negatives.

#### 2.2.6. Precision (*P*)

When it comes to the classification task, the precision represents the probability of a correct positive determination in each category and is used to reflect whether the model’s disease detection is precise, as shown in Equation (6).

(6) $$P=\frac{T_{P}}{T_{P}+F_{P}}\times 100\%$$

where *T_P_* denotes the number of true positives and *F_P_* denotes the number of false positives.

### 2.3. Analysis and Comparison of Model Results

To verify the recognition capability of AMDFNet, we conducted a comparison prediction test with appeal on six models under identical experimental conditions. The experimental results are shown in Table 1.

Table 1 compares the convolutional neural networks proposed in this paper with the classical classification network and the recently proposed plant disease classification network, and the experimental results show that the developed network has a greater advantage in terms of the number of model parameters, computational effort, and accuracy. Three fully connected layers in VGG19 increase the model’s computation and parameter sizes, making the model more susceptible to background noise and overfitting because it treats background features as classification features. As a result, VGG19 has poor generalization for disease classification in complex backgrounds. The classification validity of the model proposed in this paper is comparable to that of Inception V3, ResNet50, and DenseNet121, but it has significantly fewer parameters and requires less computational work. The proposed model has around 25% fewer parameters than ResNet50, which has the most parameters, and half as many FLOPs as DenseNet, which has the fewest parameters. It also has the highest recall of any model. The model proposed in this study uses fewer parameters and computation to achieve results comparable to those of the larger model, which lowers the model’s requirements and makes the model deployment easier. The proposed convolutional neural networks significantly increase the correct rate of disease classification in complex backgrounds by 2% on average while lowering the computational effort and inference time when compared to GoogLeNet, DICNN, and other models with a comparable number of parameters as AMDFNet. In summary, the proposed network was ranked second in terms of correctness and precision on the *C. oleifera* disease dataset; recall reached 84.95%, surpassing all models. Additionally, the proposed network achieves the lowest number of model parameters, computation, and inference time, and has great advantages in classifying *C. oleifera* diseases in complex backgrounds.

**Figure 2:**
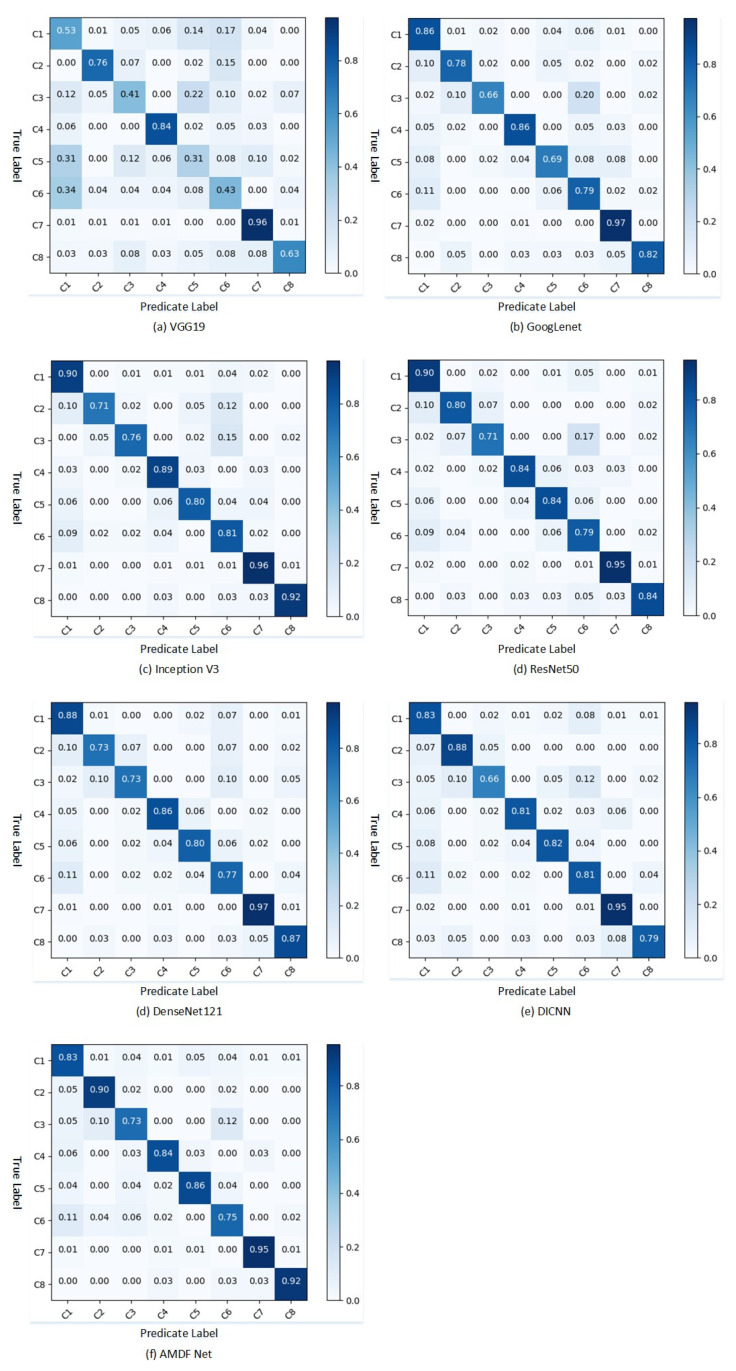
Confusion matrices of seven models. Where C1, C2, C3, C4, C5, C6, C7, and C8 represent healthy leaves, red leaf spot, algal leaf spot, tea sooty mold disease, tea round disease, tea anthracnose disease, tea white scab disease, and soft rot disease.

By using the confusion matrix approach, the performance of the above seven models was compared and tested, and the global feature fusion network was further investigated. In the confusion matrix, each row represents an actual category and each column represents the category it is predicted to be. The confusion matrix’s diagonal values represent the model’s likelihood to categorize each category correctly, whereas the values in each row represent its propensity to predict each type of input. Figure 2 depicts the confusion matrix for the above seven models.

Figure 2 shows that AMDFNet has a higher overall accuracy than the other models, with all categories correct at roughly 85% or above aside from tea anthracnose disease in category 6 and algal leaf spot in category 3. All categorization models suffered from the issue of overlap between these two categories. Upon analyzing the image dataset, it was discovered that during the later stages of widespread infection of algal leaf spot, the lesions tend to blend together with the neighboring ones, resulting in the formation of larger patches around the leaf area. These patches exhibit similar characteristics to the lesions caused by tea anthracnose disease, thus making it easier for the model to confuse them.

The model proposed in this paper obtained better classification results compared to other models in the classification of class 2 red leaf spot, class 5 tea round disease, class 7 tea white scab disease, and class 8 soft rot disease. The disease features of red leaf spot in class 2 are comparable to the feature of the land, which are readily mistaken by other models, as can be seen by examining the image data. Furthermore, AMDFNet retains some features from the small field of view through multidimensional feature fusion, making it easier to classify the disease spots in categories 5, 7, and 8, which are mostly round and small in shape and are easily missed by the convolution kernel with a larger field of view at the back of the common model.

### 2.4. Ablation Experiment

To better demonstrate the effectiveness of the multidimensional feature fusion and attention modules, we conducted ablation experiments on a self-built dataset.

Firstly, the basic Inception structure with fusion using Add is compared with the MDF fusion structure proposed in this paper. Since the Inception structure performs dimensionality reduction for all branches in each layer, it increases the number of parameters of the model as well as the computational effort. In contrast, MDF moves the fusion dimensionality reduction module after the concat operation, which can save hardware resources. Since the Add operation does not expand the number of channels, using Add fusion is the least number of parameters and computation among the three methods, but since Add fusion is a lossy fusion compared to other fusion methods, the accuracy of the model is not as good as other methods. Meanwhile, Table 2 shows the performance differences between the MDF structure and the AMDF structure. The addition of the attention structure substantially improves the performance of the MDF, proving that the attention mechanism can effectively filter out non-essential features from the fused features and improve the feature extraction capability of the model.

### 2.5. Attention Visualization

**Figure 3:**
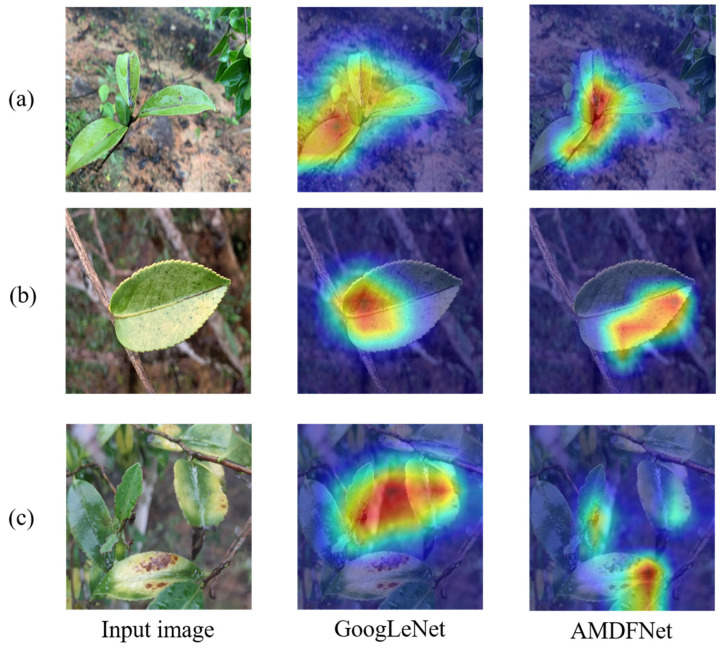
Class activation map of GoogLeNet and AMDFNet. There are three classes based on GoogLeNet focus: (**a**) GoogLeNet focuses on the correct disease area; (**b**) GoogLeNet is not affected by background noise but focuses on the wrong region; (**c**) GoogLeNet is affected by the complex background and focuses on the background region.

To further explore the effects of multidimensional attention fusion and parallel attention modules on model attention, this paper visualizes the features of AMDFNet and Inception structures using Grad-CAM [25]. GoogLeNet was used, which consists of only the basic Inception structure, as a representative of the Inception structure. The results are shown in Figure 3, and the experimental results can be roughly classified into three categories depending on the regions of GoogLeNet attention.

As shown in Figure 3a, the Inception structure has a certain ability to extract disease features in the foreground when the background is more homogeneous, but the range is not precise enough, whereas AMDFNet can further strengthen the ability to extract features and improve the accuracy of extracted features. Benefiting from the attention mechanism and multidimensional feature fusion, AMDFNet can strengthen the disease features and focus more accurately on small target features, as shown in Figure 3b; AMDFNet classifies based on black disease spots, whereas GoogLeNet focuses more on the locations with fewer disease spots. Figure 3c shows that when the background environment is more complex, GoogLeNet is seriously affected by the background noise, and the attention activation is partially focused on the image background, which easily causes classification errors. AMDFNet can resist the interference of background noise and can extract feature more accurately in the complex environment.

AMDFNet has greater interference resistance because it fuses features of various dimensions in a novel manner, enriching the features gathered at each layer while controlling the size of the model parameters. Additionally, the attention module further improves the extracted features so that they are better selected and the noisy features are effectively suppressed, increasing the accuracy of the model’s attention.

## 3. Materials and Methods

### 3.1. Data Acquisition and Preprocessing

**Figure 4:**
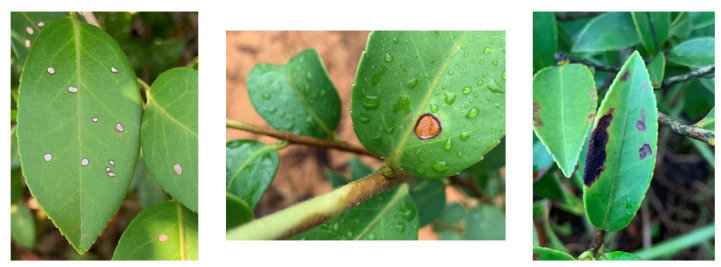
Examples of cleaned images of the collected *Camellia oleifera* disease leaf image samples, from left to right: tea white scab disease, soft rot disease, and tea anthracnose disease leaves.

The dataset employed in this study is a self-built dataset which comprised images of leaf samples representing seven prevalent *C. oleifera* diseases, alongside a category representing healthy leaves. The seven disease categories encompass red leaf spot (caused by fungus *Phyllosticta theicola* Petch), algal leaf spot (caused by the fern *Cephaleuros parasiticus*), tea sooty mold disease (caused by fungus *Neocapnodium theae* Hara), tea round disease (caused by fungus *Cercospora theae*(Cav.) Breda), tea anthracnose disease (caused by fungus *Colletotrichum gloeosporioides* penz.), tea white scab disease (caused by fungus *Phyllosticta theaefolia* Hara), and soft rot disease (caused by fungus *Agaricodochium camelliae*). The images were collected from the planting base in Hangzhou, Zhejiang Province, and the research site in Ji’an, Jiangxi Province. *C. oleifera* diseases pictures were collected under varied weather and light conditions using mobile phones for different perspectives, with the background of the photographs being land or other plants, taking into account the practical application scenarios and generality of the model. The collection period selected was between 8 a.m. and 5 p.m. on sunny days and 9 a.m. to 4 p.m. on rainy days to ensure that the disease symptoms were clearly evident. All images were captured using the automatic exposure mode and subsequently underwent data cleaning via clustering to eliminate anomalies and excessive similarity [26]. This process yielded a total of 1764 images, which were then transferred to the computer in JPG format. The cleaned images of the collected dataset are shown in Figure 4.

Due to the uneven amount of data for each disease of *C. oleifera*, it was necessary to prevent the model from learning a priori information such as the proportion of samples in the training set and thus biasing the prediction towards the majority class, as well as to avoid overfitting owing to insufficient data [27]. As a result, the data must be expanded. In this paper, data augmentation on acquired *C. oleifera* disease image data was obtained using a number of offline augmentations such as image flip, image rotation, image clipping, and Cutout. Furthermore, to avoid the effect of image size on classification, all the images were scaled to 224 × 224. The specific methods of image enhancement are as follows.

- Image scaling: scale the image to the specified size.
- Image flipping: random horizontal and vertical flipping of images.
- Image rotation: rotate images at random angles.
- Image clipping: random cropping of the input image to segment multiple disease features in a single image into multiple images.
- Cutout: randomly masking out square regions of the input image.

**Figure 5:**
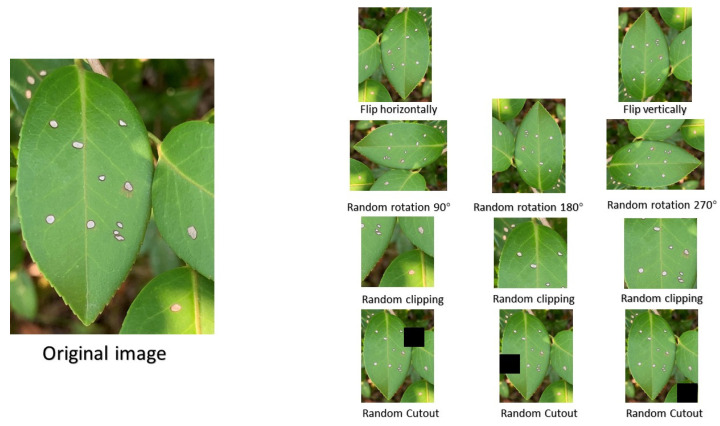
The examples of image augmentation.

Figure 5 shows an example diagram employing the above picture augmentation method, and Table 3 shows the outcomes of image augmentation. Image augmentation allows a small bit of data to have the same value as larger data without requiring a significant increase in data. Given that deep learning demonstrates enhanced performance when trained with a minimum of thousand images per class [28], along with the intricate background of this particular task, we expanded each class to encompass approximately 1500 images. A total of 20% of the experimental data are utilized for testing, 20% for validation, and 60% are used for training.

As shown in Table 3, the self-constructed database has a total of 9173 images. The database for this paper includes seven common diseases and healthy leaves of *C. oleifera*, with roughly the same number of entries in each category in an expanded database.

### 3.2. Image Recognition Model for Camellia oleifera Diseases

Convolutional neural networks have become a common deep learning method in the field of computer vision due to their ability to automatically learn and classify features from images. This approach not only reduces the number of parameters and computational effort by sharing parameters and local connections but also alleviates the overfitting caused by too many parameters, making the model easier to be trained [29]. A classical convolutional neural network generally consists of a convolutional layer, a pooling layer, and a fully connected layer. The convolutional layer is used to extract features from the input image, mapping the original data to the hidden feature space, and the pooling layer compresses the extracted features and is used to compress the data and reduce the number of parameters. The fully connected layer is used to combine the features extracted by the convolutional layer and correspond them to the sample label space, which serves as a classification.

The convolutional layer performs weight sharing through convolutional kernels, and the calculation formula is shown in Equation (7)

(7) $$x_{j}^{l}=f\left(\sum_{i\in M_{j}}x_{i}^{l-1}k_{ij}^{l}+b_{j}^{l}\right)$$

where *l* represents the ordinal number of the layers in the model, *i*, *j* represent the ordinal number of the neuron, $x_{j}^{l}$ represents the output of the *j*th neuron in the lth layer, *M_j_* represents the input feature map of the jth neuron, $k_{ij}^{l}$ represents the value of the convolution kernel of the ith and jth neurons in the lth layer, $b_{j}^{l}$ represents the bias term of the jth neuron in the lth layer, and *f* (•) is the nonlinear activation function. 

#### 3.2.1. Convolutional Neural Networks (CNNs)

Convolutional neural networks are commonly used in agriculture due to their ability to learn and extract features from input images, reduce model parameters through weight sharing and local connections, and their computational efficiency compared to standard image classification algorithms. The landmark CNN model, AlexNet, enhances the model’s convergence speed through the utilization of the ReLu activation function. It mitigates overfitting by employing the Dropout mechanism and a data enhancement strategy. Furthermore, it suggests training the model on multiple Graphics Processing Units (GPUs) to expedite the training process [30]. The Visual Geometry Group (VGG) networks optimize computational resources by utilizing multiple small convolutional kernels instead of larger ones. They also validate that deepening neural networks significantly enhance network classification performance [31]. Subsequently, the Inception framework was introduced to mitigate the problem of gradient vanishing. This was achieved by expanding the network architecture, convolving and stitching the same input using convolutional kernels with varying fields of view, rather than relying solely on an excessively deep network [32,33]. ResNet effectively addresses the problem of gradient vanishing by employing skip connections. These connections circumvent the issue of the backpropagation gradient approaching zero when the neural network’s depth becomes excessively large, thereby mitigating the challenge of training the shallow parameters [34]. Building upon this concept, densely connected convolutional networks (DenseNet) introduce dense connectivity. This approach facilitates feature reuse by establishing skip connections between all preceding and subsequent layers, thereby enabling the model to achieve superior performance with fewer parameters [35].

**Figure 6:**
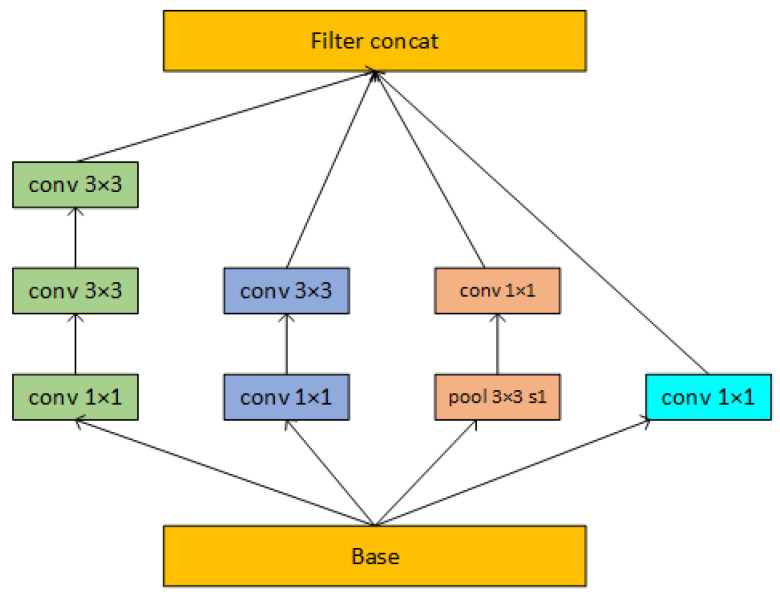
Structure of the basic Inception module.

In a comprehensive comparison, AlexNet and VGG Networks use larger convolutional kernels and more full connections, which results in a larger model volume and less effective model extraction. Through skip connection, ResNet and DenseNet may execute feature reuse well; however, excessive splices force data replication, which greatly raises FLOPs. Although the Inception structure performs slightly worse than DenseNet at extracting features, it has better perception of target features with smaller image scales due to the combination of features at different scales and is more suitable for the classification of classes with small feature disparities [36]. The Basic Inception structure is shown in Figure 6.

#### 3.2.2. Multi-Feature Fusion Block

The two main types of traditional feature fusion are early fusion, which is performed on image features, and late fusion, which is performed on prediction scores. Concat (concatenate operation) and Add are frequent procedures for early fusion. Add operations add a priori information to each channel in the feature map by directly summing the corresponding elements of the feature map. Concat, on the other hand, directly stitches multiple feature maps over the channels and will significantly increase the number of channels. The formulae for the add operation and concat operation are given in Equation (8).

(8) $$\begin{array}{cc} Z_{add} & =\sum_{i=1}^{c}\left(X_{i}+Y_{i}\right)*K_{i}\\ & =\sum_{i=1}^{c}X_{i}*K_{i}+\sum_{i=1}^{c}Y_{i}*K_{i}\\ Z_{concat} & =\sum_{i=1}^{c}X_{i}*K_{i}+\sum_{i=1}^{c}Y_{i}*K_{i+c} \end{array}$$

where *Z* represents the result after fusion and convolution, *X*, *Y* represent the input features, respectively, *c* represents the number of channels of the input features, *K* is the convolution kernel, *i* represents the *i*-th channel of the corresponding structure, and ∗ represents the convolution operation.

**Figure 7:**
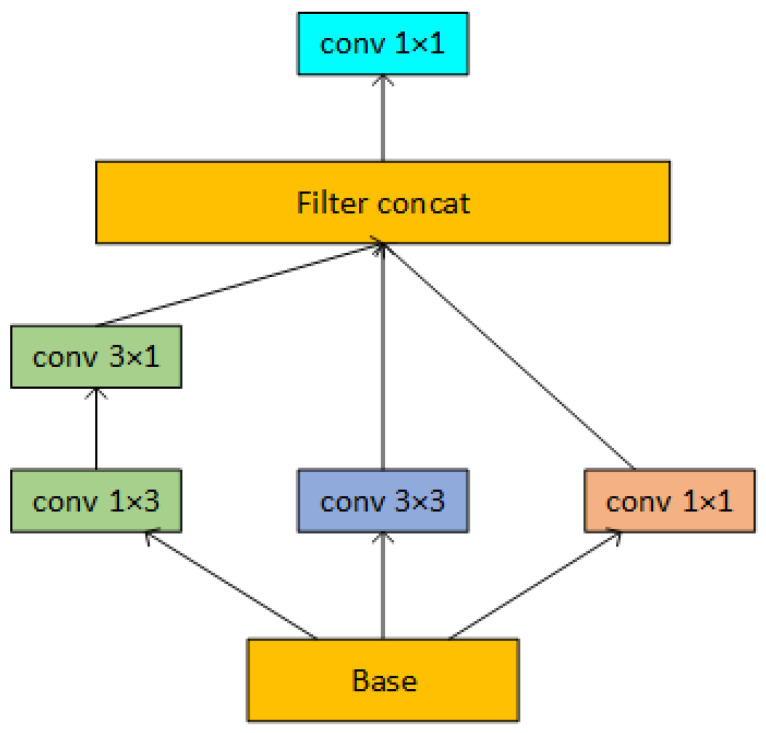
Structure of the MDF block. The feature map will have three times as many channels as Base after concat, and after 1 × 1 convolution, the number of channels will be downscaled to Base’s original size.

Compared with the Add operation, concat has no restriction on the semantic similarity of the input features and allows the number of channels of the input features to be different, which can maximize the information of the input features to be retained. However, keeping all channels will result in a much greater subsequent convolution computation and use up excessive amounts of memory and processing resources. In contrast to GoogLeNet, we place the 1 × 1 convolution kernel after the concat structure to boost the number of channels in the middle layer and enhance the information-extracting capabilities of multidimensional convolution. The 1 × 1 convolution is then used to filter and downscale the stitched features to remove extraneous data and lower the subsequent computing cost after the multi-dimensional features have been concatenated. Although two asymmetric convolutions could theoretically be equivalent to one standard convolution of the same size since the intrinsic rank of the kernel under deep learning would be more complex [37]. Considering the number of parameters in the model, we use asymmetric convolutions to replace two 3 × 3 convolutions to provide additional semantic information. We call this structure, which is seen in Figure 7, the Multi-Dimensional Fusion (MDF) block.

#### 3.2.3. Attention Mechanism Module

Attention mechanism has been popular in the field of deep learning in recent years [38]. The attention mechanism was first used to model the importance of features in image classification tasks by mimicking how people execute picture recognition, selectively ignoring some features to improve accuracy. Subsequently, the attention mechanism became the focus of research in a variety of domains. Today’s attention mechanism is mainly divided into two categories: soft attention and hard attention. Hard attention directly selects image pixel regions for attention through cropping and segmentation. However, since cropping is not differentiable, the training process is often performed through reinforcement learning, making it more difficult to train. Soft attention, on the other hand, is weighted by successive distribution intervals for regions or channels of interest. As this type of attention mechanism is differentiable, the parameters can be trained by forward propagation and backpropagation. Depending on the domain of interest of the model, soft attention can be summarized in two categories:

1. Spatial attention, by obtaining spatial information from the original image, generating a weighted mask for each location, and weighting the output with a spatial transformation module to enhance specific regions of interest.
2. Channel attention, focusing on the correlation between channels. Channel attention improves the differentiation of each channel feature by one-dimensioning the input feature map and then learning the weights of each channel using a multilayer perceptron.

**Figure 8:**
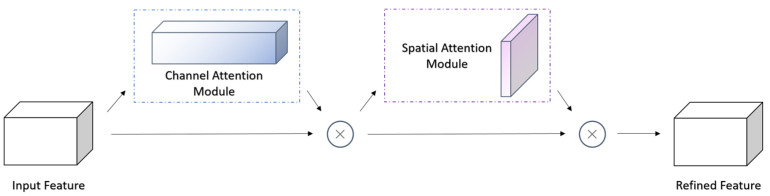
Structure of CBAM module.

SENet, which consists mostly of three operations: squeeze, excitation, and reweight is a representative channel attention mechanism [39]. It can calculate the weights between different channels and change the original feature map. In addition, it can combine the two dimensions of channel attention and spatial attention to perform adaptive feature refinement on the input feature map [40]. CBAM improves recognition accuracy by building channel attention and spatial attention tandem end-to-end modules, which the attention calculation process is shown in Equation (9), and the structure is shown in Figure 8.

(9) $$\begin{array}{c} F_{1}=M_{c}\left(F\right)⊗F\\ F_{2}=M_{s}\left(F_{1}\right)⊗F_{1} \end{array}$$

where *F_i_* is the feature map of each stage, *M_c_*(·) stands for generating channel attention, *M_s_*(·) stands for generating space attention, and ⊗ represents the multiplication of corresponding elements.

**Figure 9:**
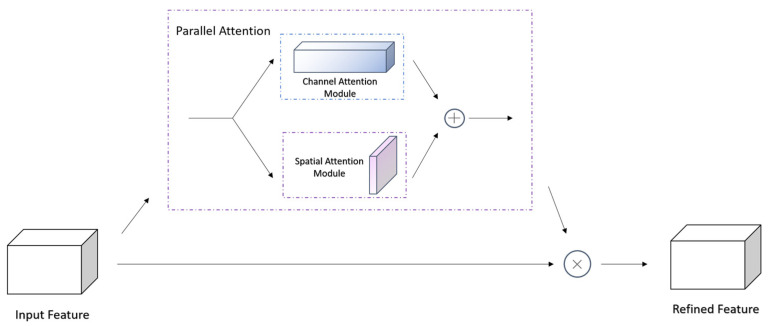
The structure of parallel attention.

Considering that channel attention and spatial attention may interfere with each other in complex backgrounds, we assemble spatial attention and channel attention in parallel and refined the feature map after superimposing the corresponding weights calculated, whose attention generation process is shown in Equation (10) and structure is shown in Figure 9.

(10) $$F_{out}=\left(M_{c}\left(F\right)⊕M_{s}\left(F\right)\right)⊕F$$

where *F_i_* is the feature map of each stage, *M_c_*(·) stands for generating channel attention, *M_s_*(·) stands for generating space attention, ⊕ represents the addition of corresponding elements, ⊗ represents the multiplication of corresponding elements.

**Figure 10:**
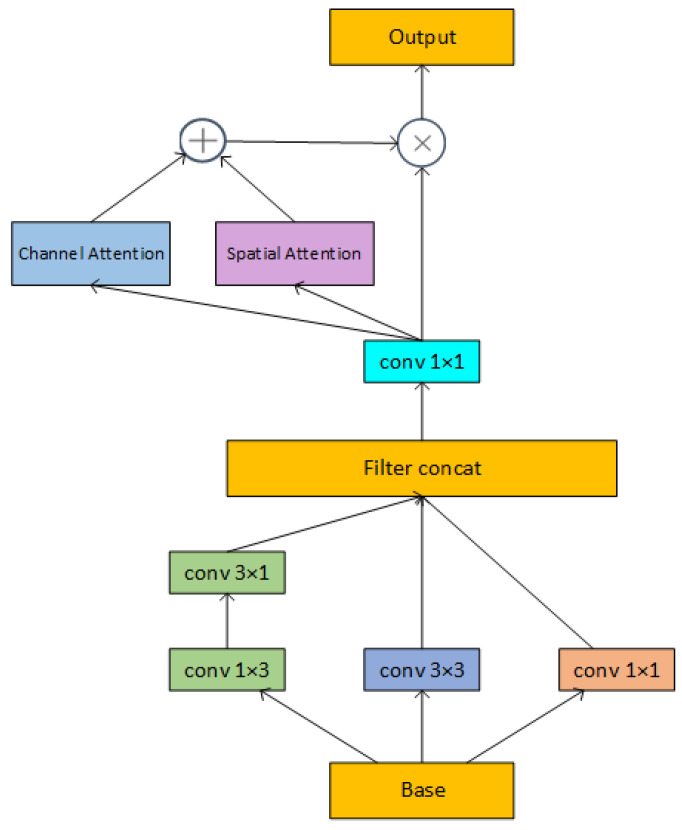
Structure of the AMDF block. At the conclusion of the original MDF module, parallel attention is provided to enhance the model feature extraction capabilities.

Parallel attention was added to the MDF module and the extraction ability of the module was enhanced by attention to the features after fusion to form the AMDF module. Figure 10 depicts the AMDF module’s structural layout.

#### 3.2.4. Attention and Multi-Dimensional Feature Fusion Neural Network (AMDFNet)

**Figure 11:**
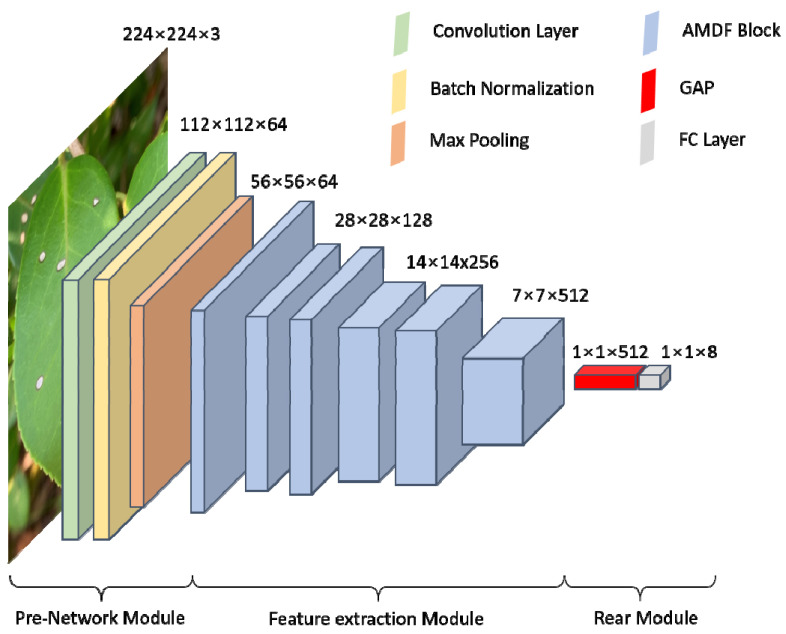
Structure diagram of the AMDF model.

The proposed method combines multidimensional feature fusion and attention mechanism for disease identification in the complex background of *C. oleifera* which includes three main modules: Pre-NetWork Module, Feature extraction Module, and Rear Module. The Pre-NetWork Module consists of a 7 × 7 convolutional kernel, BatchNormal layer, and Max pooling for initial extraction of the input image to obtain as much semantic information as possible. The Feature extraction Module consists of several AMDF blocks with different numbers of channels. Considering the extraction capability and model size, it was used one 64-dimensional, two 128-dimensional, two 256-dimensional, and one 512-dimensional AMDF blocks in series to further extract the input image features. The Rear Module is mainly used to classify the extracted features and score each class by global average pooling (GAP) layer and fully connected (FC) layer to obtain the classification results. The specific structure of the model is shown in Figure 11 and Table 4.

## 4. Discussion

The appearance features of *C. oleifera* diseases in actual environments exhibit various states at various periods, leading to significant variations in photographs of the same disease at various times; conversely, some different kinds of diseases exist with similar image features at various times. The multidimensional feature fusion structure can integrate the perceptual domains of convolution kernels on different dimensions to increase the model’s ability to identify categories with similar picture features, whereas traditional classifiers are poor at doing so [41]. In the original work, the feature maps are first reduced to different dimensions and then extracted and stitched together, which may lead to differences in the extracted feature maps in different dimensions. In contrast, the method proposed directly extracts and stitches the original feature map in different dimensions and then reduces the dimensionality of the stitched features, which not only reduces the computational effort caused by multiple dimensionality reduction operations but also maximizes the adequacy of the original feature map extraction.

Unlike images of plant leaf diseases acquired in the laboratory, images acquired in the field not only do not have uniform lighting and angles, but also the complex background environment introduces a large number of noisy features, making feature extraction more difficult. The attention mechanism can compute weights for various features while employing soft thresholding methods to reduce noise interference on features. The parallel spatial attention and channel attention used in this study allows the fused features to be readjusted while filtering the background noise, amplifying feature differences, and reducing the classification difficulty of the classifier. Although in previous studies, the method of serializing spatial attention and channel attention has worked slightly better than parallel attention mechanisms [39]. However, considering that in this experiment, a 1 × 1 convolutional kernel has been used to combine features under different fields of view, both channel attention and spatial attention can provide better attention weights. Therefore, in order to prevent the two attention structures from interfering with each other and to reduce the training difficulty, spatial attention and channel attention were used in parallel, calculated, and assigned independently, making the training process smoother and easier to converge.

The current work successfully implements a lightweight model to classify *C. oleifera* disease images in complex environments, with the help of Inception’s multi-branch structure for feature fusion to obtain multidimensional features, and an attention mechanism for noise reduction and feature enhancement. It makes it possible to use a mobile device equipped with an image classification model for disease detection in a realistic environment. Compared to existing models, the proposed model has better performance in complex environments and also has lower requirements for running devices, reflecting higher advantages. However, the deep learning model proposed in this study is still not free from the limitation of data volume and needs to learn high-dimensional features from a large amount of image data in order to obtain correct identification capability. In this study, only a few common diseases on *C. oleifera* leaves were sampled, which limits the ability of the model to classify more rare diseases. In future research, combining easily accessible classification knowledge such as text or knowledge graphs should be considered to guide the classification ability of images, using a small number of samples to complete the training of the classifier and reducing the data volume requirement of the model. Even so, by comparing the performance and consumption of classical classification models and AMDFNet, AMDFNet can replace expert advice in complex environments and provide credible advice for the diagnosis of common *C. oleifera* diseases. The method can be used in specific agricultural production for decision support in integrated disease management.

## 5. Conclusions

In this paper, a *C. oleifera* disease identification model based on multidimensional feature fusion and attention mechanism is proposed. The AMDF structure is proposed by the fusion operation of the branches of the Inception structure to obtain multidimensional features and feature enhancement by the attention mechanism. It improves the classification ability of the model in complex backgrounds while reducing the number of model parameters and computational effort. The training was performed on the ImageNet dataset and migrated to the self-built *C. oleifera* dataset for testing. The test results showed that the correct rate was improved by 2.3% compared to GoogLeNet using only the Inception structure, whereas the number of parameters was reduced to a quarter compared to large models such as ResNet50 with little difference in correct rate.

The CNN model proposed in this paper can identify *C. oleifera* diseases quickly and accurately and provides a feasible solution for identifying selected leaf diseases of this species. All data in this paper were collected in a real environment. In the future, the disease identification model will be improved by adding other common and relevant disease symptoms. The simplified model can also be deployed on mobile for easy use by farmers.

## Figures and Tables

**Table 1:** Comparison of the test result of *Camellia oleifera* disease identification models. The best case scenarios for each indicator are highlighted in bold.

Model	*Ts* (s)	*Params* (MB)	*FLOPs* (GB)	*Acc* (%)	*R* (%)	*P* (%)
VGG19	0.278	532.54	39.3	68.01	61.76	62.90
GoogLeNet	0.254	21.45	3.0	84.48	80.37	83.38
Inception V3	0.275	83.30	11.4	**87.73**	84.33	**86.75**
ResNet50	0.265	89.94	8.2	86.29	83.48	84.29
DenseNet 121	0.263	26.88	5.7	86.21	82.67	83.82
DICNN	0.271	**15.01**	6.2	84.86	81.89	83.74
AMDFNet	**0.250**	23.21	**2.7**	86.78	**84.95**	84.61

**Table 2:** Comparison of AMDFNet ablation experiments by module. The best case scenarios for each indicator are highlighted in bold.

Model	*Ts* (s)	*Params* (MB)	*FLOPs* (GB)	*Acc* (%)	*R* (%)	*P* (%)
Basic Inception Structure	0.259	23.70	3.4	75.86	70.53	73.60
Inception Structure + Add fusion	**0.250**	**17.85**	**2.2**	75.67	69.36	73.31
MDF Structure	**0.250**	22.80	2.6	82.37	79.60	77.86
AMDF Structure	**0.250**	23.21	2.7	**86.78**	**84.95**	**84.61**

**Table 3:** Number of images of various types of *Camellia oleifera* diseases before and after data augmentation.

Disease	Original Numbers	Expanded Numbers
Tea anthracnose disease	183	1473
Soft rot disease	127	1554
Red leaf spot	137	1481
Tea sooty mold disease	219	1540
Tea white scab disease	514	1594
Tea round disease	163	1531
Algal leaf spot	142	1542
Healthy leaves	279	1455

**Table 4:** Composition of the AMDF model.

Type	Kernel Size/Stride/Padding	Output Size
Convolutional Layer	7 × 7/2/3	112 × 112 × 64
Max Pooling	3 × 3/2/1	56 × 56 × 64
AMDF Block		56 × 56 × 64
AMDF Block		28 × 28 × 128
AMDF Block		28 × 28 × 128
AMDF Block		14 × 14 × 256
AMDF Block		14 × 14 × 256
AMDF Block		7 × 7 × 512
GAP		1 × 1 × 512
FC Layer		8

## Data Availability

Not applicable.
